# microRNA Expression and Its Association With Disability and Brain Atrophy in Multiple Sclerosis Patients Treated With Glatiramer Acetate

**DOI:** 10.3389/fimmu.2022.904683

**Published:** 2022-06-14

**Authors:** María I. Dominguez-Mozo, Ignacio Casanova, Laura De Torres, Yolanda Aladro-Benito, Silvia Perez-Perez, Angel Garcia-Martínez, Patricia Gomez, Sara Abellan, Esther De Antonio, Carlos Lopez-De-Silanes, Roberto Alvarez-Lafuente

**Affiliations:** ^1^ Research Group in Environmental Factors of Neurodegenerative Diseases, Health Research Institute Hospital Clínico San Carlos (IdISSC), Madrid, Spain; ^2^ Department of Neurology, Hospital Universitario de Torrejón, Madrid, Spain; ^3^ School of Medicine, Universidad Francisco de Vitoria, Madrid, Spain; ^4^ Department of Neurology, Hospital Universitario de Getafe, Madrid, Spain; ^5^ Department of Radiology, Hospital Universitario de Torrejón, Madrid, Spain

**Keywords:** multiple sclerosis, microRNA, biomarker, cognitive dysfuction, brain atrophy

## Abstract

**Background:**

MicroRNAs are small non-coding RNA that regulate gene expression at a post-transcriptional level affecting several cellular processes including inflammation, neurodegeneration and remyelination. Different patterns of miRNAs expression have been demonstrated in multiple sclerosis compared to controls, as well as in different courses of the disease. For these reason they have been postulated as promising biomarkers candidates in multiple sclerosis.

**Objective:**

to correlate serum microRNAs profile expression with disability, cognitive functioning and brain volume in patients with remitting-relapsing multiple sclerosis.

**Methods:**

cross-sectional study in relapsing-remitting multiple sclerosis patients treated with glatiramer acetate. Disability was measured with Expanded Disability Status Scale (EDSS) and cognitive function was studied with Symbol Digit Modalities Test (SDMT). Brain volume was analyzed with automatic software NeuroQuant^®^.

**Results:**

We found an association between miR.146a.5p (r_s_:0.434, p=0.03) and miR.9.5p (r_s_:0.516, p=0.028) with EDSS; and miR-146a.5p (r_s_:-0.476, p=0.016) and miR-126.3p (r_s_:-0.528, p=0.007) with SDMT. Regarding to the brain volume, miR.9.5p correlated with thalamus (r_s_:-0.545, p=0.036); miR.200c.3p with pallidum (r_s_:-0.68, p=0.002) and cerebellum (r_s_:-0.472, p=0.048); miR-138.5p with amygdala (r_s_:0.73, p=0.016) and pallidum (r_s_:0.64, p=0.048); and miR-223.3p with caudate (r_s_:0.46, p=0.04).

**Conclusions:**

These data support the hypothesis of microRNA as potential biomarkers in this disease. More studies are needed to validate these results and to better understand the role of microRNAs in the pathogenesis, monitoring and therapeutic response of multiple sclerosis.

## Introduction

MicroRNAs are promising biomarkers in multiple sclerosis (MS). They are endogenous, non-coding RNA particles, between 20 and 25 nucleotides in length that regulate gene expression at a post-transcriptional level, by blocking translation or inducing the degradation of messenger RNA (1). It is estimated that up to one third of the human genes are regulated by these miRNAs (2). They participate in multitude of cellular processes and, thus, they could play important roles in several mechanisms of MS such as remyelination, neurodegeneration, autoimmunity or blood brain barrier homeostasis (3, 4). Over the last years, dysregulated miRNA function and patterns of expression has been demonstrated in MS patients compared to healthy subjects (5–7) and in different aspects of the disease: relapses versus remission (8), different clinical phenotypes (9, 10), radiological patterns (11) and even treatment effects (12–14). Moreover, they can be easily, repeatedly, reliably and non-invasively measured in different samples. For these reasons they are promising candidates to become clinically useful biomarkers to monitor the progression of the disease and as predictors of the therapeutic response to disease-modifying treatments (15, 16)

Less information is available regarding the use of miRNAs to monitor clinical evolution such as disability worsening measured with EDSS (17), or progression of non-motor symptoms like cognitive impairment (18, 19). This complication is a frequent manifestation of MS, even in early stages of the disease (20–22), with a high impact on the overall clinical situation of these patients (23). Symbol Digit Modalities Test (SDMT) is the most recommended screening test to monitor for this complication in MS (24).

For these reasons, in this study we aimed to investigate the correlation between serum miRNAs profile expression and clinical disability, cognitive functioning and brain volume in patients with remitting-relapsing multiple sclerosis treated with glatiramer acetate, in order to increase the knowledge of the relationship between microRNA and the whole MS clinical and radiological spectrum.

## Material and Methods

### Study Design

Cross-sectional study in a cohort of MS patients attending the demyelinating diseases unit at the Hospital Universitario de Torrejon and Hospital Universitario de Getafe in Madrid, Spain, from September/2016 to September/2017. We selected relapsing remitting multiple sclerosis patients (RRMS) according to McDonald 2010 criteria (25), on stable treatment with glatiramer acetate (GA) during at least 6 months. GA was selected to try to have the most possible homogeneous sample. In the first hand we decided to choose first line treatment patients. Normally these patients represent the early phase of MS with less time of evolution of MS and fewer therapies, which would reduce the possible changes of miRNA expression due to longer durations of the disease or associated with previous treatments. Regarding first line drugs, GA pharmacokinetics could be associated with fewer metabolic changes and therefore with less changes of miRNA profiles not directly related with the mechanism of action of the drug (26, 27). Exclusion criteria were: secondary progressive multiple sclerosis (SPMS) and primary progressive multiple sclerosis (PPMS) according to Lublin 2013 phenotypes classification (28), relapse or corticosteroids treatment in the last 3 months previous to the study, and any contraindication to perform MRI. All patients were prescribed GA in accordance with Spanish Society of Neurology clinical practice guidelines that include protocols about using disease modifying treatment and monitoring its effects (29). All patients gave their consent to participate in the study. The study complied with the Helsinki declaration (30), and was approved by the ethical committee of the Hospital Universitario de Getafe and by the Spanish Agency of Drugs and Health Products (code CLD-GLA-2017-01).

Sex, age at disease onset, age at GA onset, age at the moment of the study and Expanded Disability Status Scale (EDSS) were collected. Cognitive function was assessed using the symbol digit modalities test (SDMT) (31).

### MicroRNA Selection and Analysis

We selected the best miRNAs candidates for RRMS and cognitive dysfunction (CD) through simple topological analysis (Anaxomics^®^). A cut-off ≥ 0,8 for the global score was established. The first 20 miRNAs met this criteria and were finally selected. First of all, the molecular characterizations of MS and CD were performed through hand‐curated evaluation of indexed scientific publications in PubMed, obtaining 293 proteins for MS and 59 proteins for CD. MicroRNAs were collected by search in the databases HMDD, miR2Disease, miRWalk 2.0, NSDNA, PhenomiR 2.0, miRdSNP and miREnvironment. These miRNAs were mapped to genes/proteins through miRTarBase, a miRNA‐protein relationship open database that stores information about experimentally validated miRNA targets. Finally three different types of scores were calculated for each miRNA to obtain the final ranking score: 1) percentage of miRNA‐disease related elements over the total of miRNA targets; 2) percentage of miRNA‐disease related elements over the total of disease effectors; and 3) Haussdorf distance between the whole set of miRNA targets and the conditions of interest. A final global score was obtained by calculating a weighted mean of the three rankings, with the percentage over the total of miRNA targets weighted twice as strongly as the other two measures (**Table 1**). Blood (10 ml) was drawn from each patient using CPT tubes (Becton Dickinson NJ, USA). Peripheral blood mononuclear cells (PBMC) were extracted after centrifugation at 2500 g during 30 minutes. RNA was isolated with the QIAmp RNA blood Mini Kit, following the manufactere’s instructions (QIAGEN, Hilden, Germany). The cDNA was obtained *via* reverse transcription with the kit multiplex RT for Taqman^®^ microRNA assays (Life Technologies, Foster City, CA). MiRNAs profile was determined with Locked nucleic acid (LNA) SYBER green-based quantitative real-time polymerase chain reaction (LNA-based qPCR) (Exiqon). Normalization was performed using the mean expression of two miRNAs: miR191-5p and miR30c-5p. The normalized cycle quantification (Cq) value was calculated as mean Cq - assay Cq.

**Table 1:** List of microRNAs included in the study after simple topological analysis (Anaxomics ^®^) ordered by rank.

microRNas	Score 1	Score 2	Haussdorf distance	Final score
199a-5p	0.96	0.93	0.99	0.96
126-3p	0.99	0.78	1.00	0.94
29b-3p	0.89	0.94	0.98	0.92
145-5p	0.89	0.90	0.99	0.92
200c-3p	0.88	0.87	1.00	0.90
143-3p	0.95	0.90	0.83	0.90
199a-3p	0.92	0.81	0.90	0.89
203a-3p	0.84	0.93	0.94	0.89
138-5p	0.91	0.69	0.99	0.88
146a-5p	0.85	0.81	0.97	0.87
200b-3p	0.84	0.81	0.99	0.87
9-5p	0.81	0.93	0.92	0.86
21-5p	0.77	0.98	0.93	0.86
29a-3p	0.80	0.87	0.98	0.86
125a-5p	0.82	0.90	0.84	0.84
223-3p	0.88	0.71	0.88	0.84
133b	0.96	0.61	0.79	0.83
204-5p	0.83	0.93	0.72	0.82
34a-5p	0.73	0.95	0.89	0.82
155-5p	0.69	0.98	0.94	0.82

Score 1: Percentage of miRNA-disease related elements over the total of miRNA targets; Score 2: Percentage of miRNA-disease related elements over the total of disease effectors.

### MRI and Brain Volume Analysis

MRI images were acquired following the MAGNIMS recommendations on the use of brain MRI in multiple sclerosis (32) with a minimum magnetic field strength of 1.5T, a maximum slice thickness of 3 mm without a gap, and the following sequences: axial pre and post-gadolinium T1-weighted, axial proton density and/or T2-weighted, and axial and sagittal T2-fluid-attenuated inversion recovery. Isovolumetric sagittal T1 (3D-SPGR) sequence was performed with the following parameters TR 1/4 8.5 ms; TE1/43.2ms; TI1/4700ms; flip angle (FA)1/412; bandwidth 1/4 31.25 kHz, to perform the volumetric analysis. Whole brain volume, grey matter volume, white matter volume, cerebellum volume, basal ganglia volume and T1 lesion load volume were obtained using the automatic software NeuroQuant ^®^.

### Statistics

Numerical variables were expressed as median and interquartile range (25th, 75th percentile), and categorical variables as percentages. Correlation between miRNAS and EDSS, cognitive status and MRI data were analyzed with Spearman correlation coefficient (r_s_). Statistical significance was set at p<0,05. Data were analyzed using the Statistical Package for Social Sciences, version 19.0 (IBM SPSS, Inc., Chicago, IL, USA)

## Results

We recruited 27 patients. Demographic and clinical data are summarized in **Table 2**. They included the typical RRMS population, with a female preference (19 female VS 8 male patients) and young onset of disease (median: 31.9 years). They also represent a typical early MS phase with early treatment initiation (median: 32.8 years at GA onset) and a mild EDSS (median: 1; interquartile range: 0-2.5).

**Table 2:** Demographic and clinical data.

SexN (F:M)	Age at MS onsetMd (ICR)	Age at GA onsetMd (ICR)	Time with GAMd (ICR)	EDSSMean ± SD
19:8	31.9 (25.1-41.9)	32.8 (26.6-44.9)	4 (2.1-6.4)	1.4 ± 1.7

N, number; F, Female; M, male; Md, Median; ICR, Interquartile range; SD, Standard Deviation; MS, Multiple Sclerosis; GA, Glatiramer acetate; EDSS, Expanded Disability Status Scale.

MRI data is represented in **Table 3**. Seven scans were unavailable due to technical incompatibility with NeuroQuant software.

**Table 3:** MRI data.

WBVMd (ICR)	CGMVMd (ICR)	WMVMd (ICR)	T1Md (ICR)	CerebellumMd (ICR)
1185.7 (1115.6-1297.3)	502.2 (462.5-544.3)	473.8 (427.2-506.8)	0.37 (0.1-0.72)	133.7 (128.1-143.9)
Thalamus Md (ICR)	Caudate Md (ICR)	Putamen Md (ICR)	Pallidum Md (ICR)	Amygdala Md (ICR)
14.5 (13.5-15.6)	6.1 (5.7-7.2)	10.6 (9.5-11.2)	1.6 (1.3-1.9)	3.4 (2.9-3.9)

WBV, whole brain volume; CGMV, Cortical grey matter volume; WMV, White matter volume; T1, T1 lesion volume; Md, Median; ICR, Intercuartile Range; Volume in ml; T1, T1 lesion volume.

We performed correlations between miRNA and sex and age. Only miR-203a.3p was correlated with age (r_s_:-0,523; p=0,026). Since this microRNA was not associated with any clinical or radiological variable we did not adjust with it for the other comparisons. None microRNA was associated with sex.

**Figure 1:**
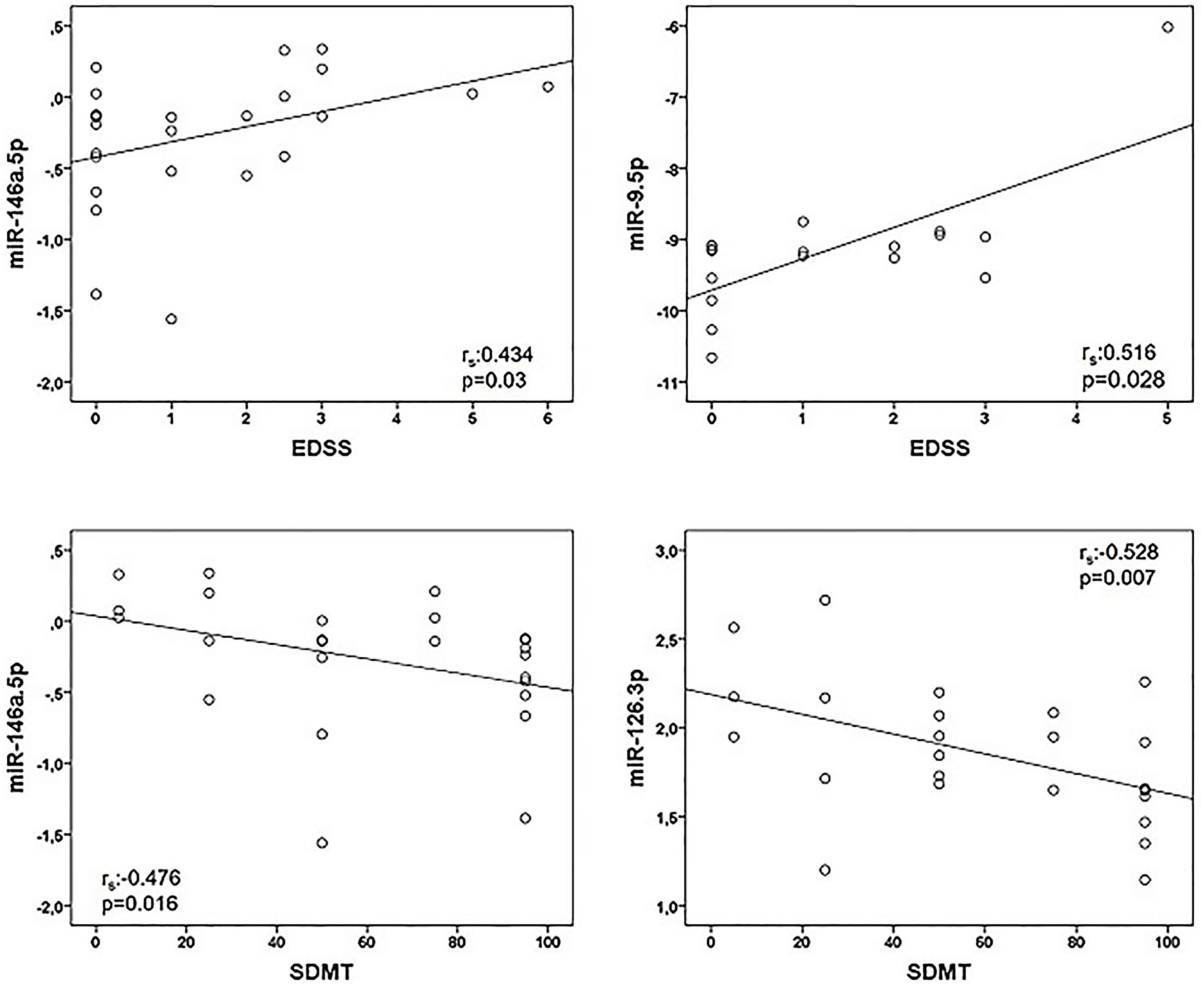
Correlations between microRNAs (miRNAs) expression levels and clinical variables in multiple sclerosis patients. Twenty-five multiple sclerosis patients were assessed in all correlations except that of miR-9-5p and EDSS (n = 18). Spearman correlation test was used for statistical analysis. EDSS, Expanded Disability Status Scale; r_s_, Spearman correlation coefficient; SDMT; Symbol Digit Modalities Test.

Correlations between miRNA and clinical data are represented in **Table 4**. We found a positive association between miR-146a.5p (r_s_:0.434, p=0.03) and EDSS, and between miR-9.5p (r_s_:0.516, p=0.028) and EDSS. Regarding the cognitive function we found a negative association of miR-146a.5p (r_s_:-0.476, p=0.016) and miR-126.3p (r_s_:-0.528, p=0.007) with SDMT (**Figure 1**), and also a trend to a negative association between miR-9.5p and SDMT (r_s_:-0.464, p=0.06). Both measures were consistent, with greater values of the miRNAs related to greater EDSS and lower SDMT punctuations.

**Table 4:** Correlations between miRNAs and EDSS and SDMT.

miRNA	EDSS (r_s_)	P	n	SDMT (r_s_)	P	n
**9-5p**	**0.516**	**0.028**	**18**	**-0.464**	**0.06**	**17**
21-5p	0.177	0.39	25	-0.22	0.3	25
29a-3p	0.213	0.31	25	-0.11	0.6	25
29b-3p	0.322	0.12	25	-0.33	0.11	25
34a-5p	0.372	0.08	23	-0.36	0.09	23
125a-5p	0.085	0.69	25	-0.12	0.57	25
126-3p	0.364	0.07	**25**	**-0.528**	**0.007**	**25**
133b	-0.09	0.97	21	0.02	0.93	21
138-5p	0.25	0.94	11	-0.06	0.86	11
143-3p	0.2	0.35	25	-0.09	0.66	25
145-5p	0.204	0.33	25	-0.24	0.25	25
**146a-5p**	**0.434**	**0.03**	**25**	**-0.476**	**0.016**	**25**
155-5p	0.102	0.63	25	0.016	0.94	25
199a-3p	0.26	0.21	25	-0.31	0.13	25
199a-5p	0.167	0.43	25	-0.27	0.19	25
200b-3p	0.083	0.71	23	-0.06	0.78	17
200c-3p	0.154	0.48	23	-0.23	0.29	23
203a-3p	-0.93	0.72	17	-0.24	0.35	17
204-5p	0.214	0.38	19	-0.04	0.86	19
223-3p	0.222	0.29	25	-0.07	0.76	25

R_s_ Spearman correlation coefficient. EDSS, Expanded Disability Status Scale; SDMT, Symbol Digit Modalities Test. Statistically significant associations are highlighted in bold.

**Figure 2:**
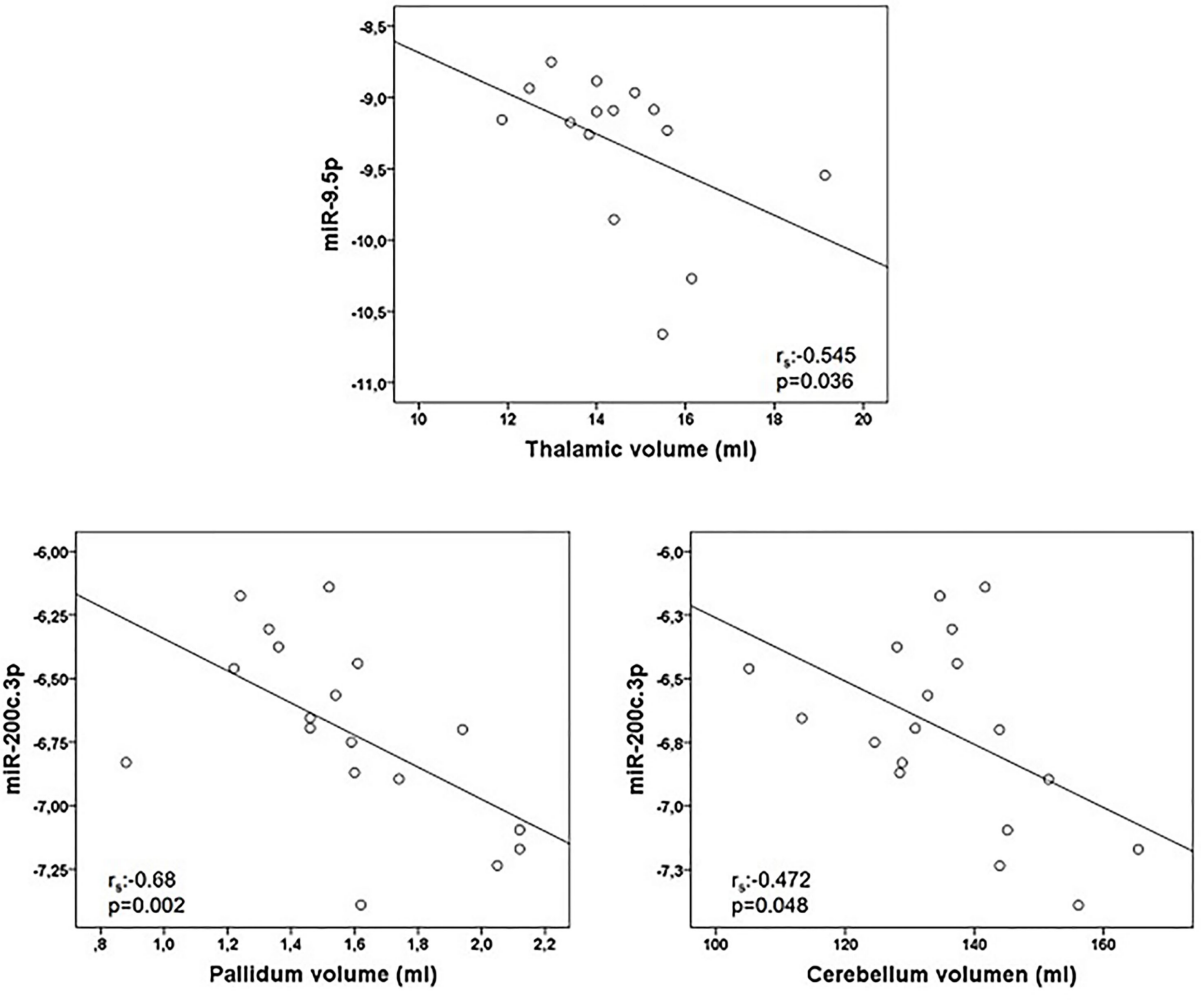
Graphical representations of microRNAs (miRNAs) expression levels associated negatively with magnetic resonance images (MRI) variables. MiR-9.5p was analyzed in 15 multiple sclerosis patients and miR-200c.3p in 18 patients. Spearman correlation test was used for statistical analysis. r_s_: Spearman correlation coefficient.

**Figure 3:**
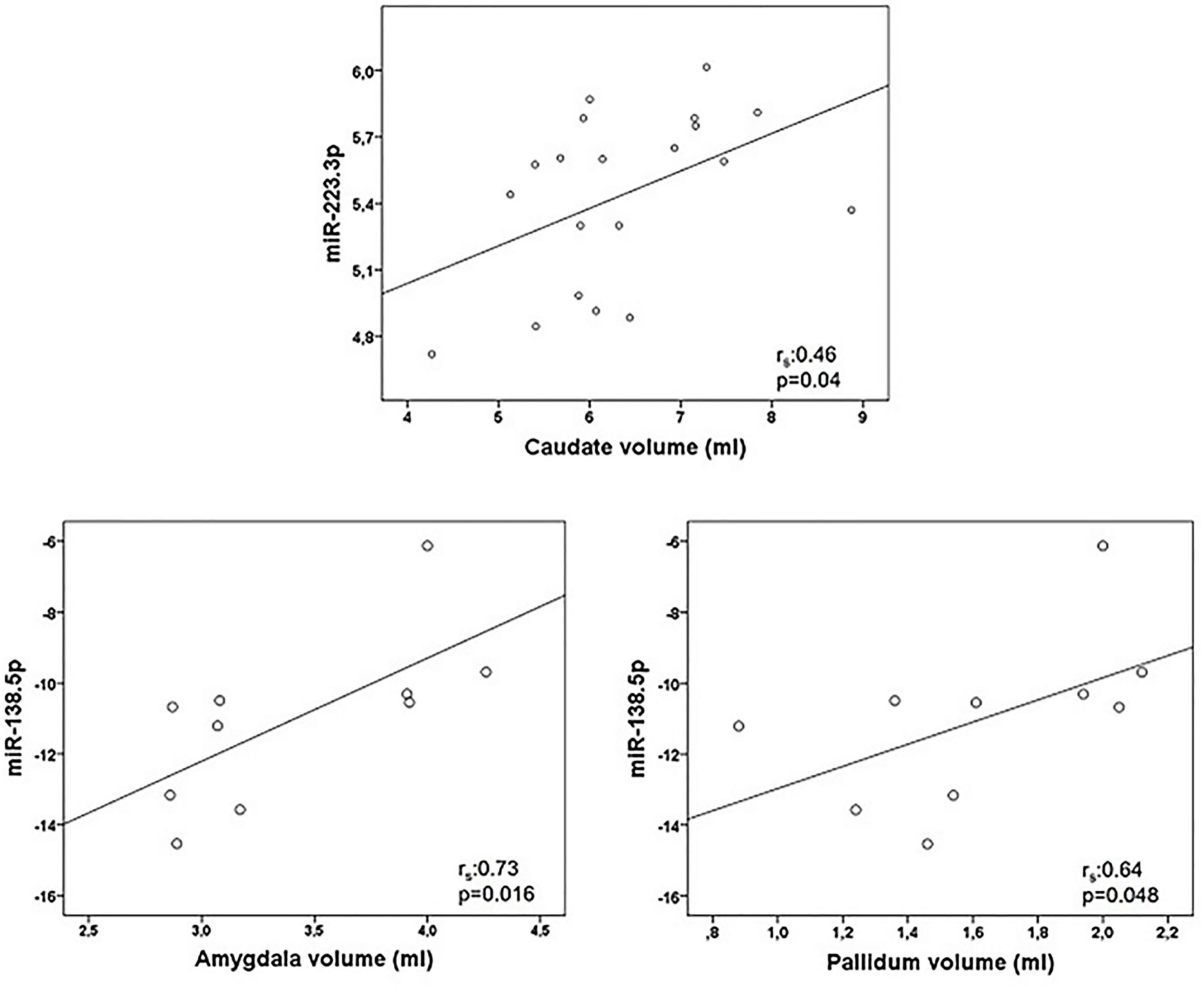
Graphical representations of microRNAs (miRNAs) expression levels associated positively with magnetic resonance images (MRI) variables. MiR-223.3p was analyzed in 20 multiple sclerosis patients and miR-138.5p in 10 patients. Spearman correlation test was used for statistical analysis. r_s_: Spearman correlation coefficient.

Correlations between miRNA and MRI data are summarized in **Table 5**. Of notice, we found a negative association between miR-9.5p and thalamic volume (r_s_:-0.545, p=0.036), and between miR-200c.3p and pallidum and cerebellum (r_s_:-0.68, p=0.002; r_s_: -0.472, p=0.048) (**Figure 2**). Again, the findings of mir-9.5p were consistent with the clinical data, with higher values of this miRNA associated with worse clinical outcomes and less thalamic volume. On the other hand, we found a positive association between miR-138.5p and amygdala and pallidum volume (r_s_:0.73, p=0.016 and r_s_:0.64, p=0.048) and between miR-223.3p and caudate (r_s_:0.46, p=0.04) (**Figure 3**). Surprisingly, we did not find any correlation between miRNAs and whole brain volume (WBV), white matter volume (WMV), cortical grey matter volume (CGMV) and hypointense T1 lesion volume (T1LV).

**Table 5:** Correlations between miRNAs and MRI.

miRNA								WBV			CGMV					WMV				T1				Cerebellum		
					n			R_s_	p		R_s_			p		R_s_		p		R_s_		p		R_s_		p
9-5p					15			-0.11	0.7		-0.05			0.86		-0.3		0.27		0.04		0.89		-0.05		0.85
21-5p					20			0.12	0.61		0.1			0.67		0.07		0.77		-0.02		0.95		0.21		0.38
29a-3p				20				0.29	0.22		0.23			0.34		0.19		0.42		-0.22		0.36		0.41		0.07
29b-3p				20				-0.14	0.56		-0.21			0.38		-0.18		0.45		-0.13		0.58		-0.05		0.82
34a-5p			18					0.03	0.91		0.03			0.89		-0.14		0.58		-0.15		0.56		0.06		0.8
125a-5p			20					0.11	0.65		0.08			0.73		0.14		0.55		-0.01		0.98		0.1		0.67
126-3p		20						-0.23	0.32		-0.22			0.36		-0.27		0.26		0.14		0.56		-0.24		0.3
133b					17			-0.19	0.46		-0.07			0.8		-0.17		0.51		-0.24		0.36		-0.16		0.54
138-5p					10			0.35	0.33		0.6			0.067		0.22		0.53		-0.36		0.31		0.29		0.42
143-3p					20			0.19	0.41		0.19			0.41		0.07		0.76		-0.35		0.13		0.36		0.12
145-5p					20			-0.02	0.95		-0.06			0.82		-0.07		0.76		-0.35		0.13		0.07		0.78
146a-5p					20			-0.12	0.6		-0.045			0.85		-0.24		0.32		-0.22		0.35		0.09		0.69
155-5p					20			0.04	0.86		0.05			0.83		-0.07		0.77		0.08		0.75		-0.86		0.72
199a-3p					20			-0.06	0.8		0.01			0.97		-0.2		0.39		-0.14		0.54		0.18		0.46
199a-5p					20			-0.01	0.97		0.04			0.87		-0.1		0.68		-0.2		0.41		0.15		0.54
200b-3p					19			0.01	0.97		0.13			0.59		-0.2		0.39		-0.07		0.77		0.04		0.88
**200c-3p**	18							-0.39	0.11		-0.33			0.18		-0.38		0.13		0.13		0.61		**0.47**		**0.048**
203a-3p	13							0.09	0.76		0.04			0.89		0.13		0.67		0.26		0.4		-0.09		0.75
204-5p	15							0.004	0.99		-0.04			0.89		0.03		0.91		0.16		0.57		0.11		0.69
223-3p	20							0.25	0.29		0.21			0.37		0.17		0.49		-0.2		0.39		0.29		0.22

miRNA							Thalamus					Caudate			Putamen				Pallidum				Amygdala			
(cont)					n		R_s_			p		R_s_	p		R_s_		p		R_s_		p		R_s_		p	
**9-5p**					15		**-0.55**			**0.036**		0.03	0.91		-0.23		0.41		-0.46		0.085		-0.2		0.47	
21-5p					20		0.12			0.62		0.33	0.16		-0.17		0.47		0.11		0.66		0.22		0.35	
29a-3p					20		0.36			0.12		0.23	0.32		0.09		0.7		0.44		0.055		0.23		0.33	
29b-3p					20		-0.28			0.24		-0.01	0.96		-0.41		0.07		0.01		0.99		-0.31		0.19	
34a-5p					18		-0.05			0.84		0.27	0.28		-0.27		0.27		-0.18		0.47		0.001		0.97	
125a-5p					20		0.07			0.76		0.25	0.29		-0.1		0.65		0.07		0.78		0.22		0.34	
126-3p					20		-0.35			0.14		0.01	0.95		-0.43		0.06		-0.31		0.19		-0.38		0.09	
133b					17		-0.19			0.45		-0.33	0.2		-0.15		0.56		-0.13		0.6		-0.14		0.59	
**138-5p**					10		0.35			0.33		0.44	0.2		0.44		0.2		**0.64**		**0.048**		**0.73**		**0.016**	
143-3p					20		0.27			0.25		0.42	0.06		0.22		0.36		0.41		0.07		0.27		0.25	
145-5p					20		0.05			0.85		0.15	0.53		0.02		0.94		0.29		0.22		0.14		0.57	
146a-5p					20		-0.24			0.3		-0.11	0.63		-0.26		0.26		0.04		0.87		-0.14		0.56	
155-5p					20		-0.09			0.69		-0.19	0.41		-0.17		0.47		-0.24		0.3		0.15		0.52	
199a-3p					20		-0.08			0.73		0.11	0.63		-0.11		0.63		0.26		0.27		0.07		0.78	
199a-5p					20		-0.15			0.5		-0.04	0.88		-0.16		0.5		0.27		0.24		0.04		0.88	
200b-3p					19		0.2			0.41		0.32	0.18		0.16		0.52		0.13		0.6		0.33		0.17	
**200c-3p**	18						-0.4			0.1		-0.2	0.42		-0.45		0.06		**-0.68**		**0.002**		-0.33		0.18	
203a-3p	13						-0.47			0.11		-0.04	0.9		-0.38		0.2		-0.36		0.22		0.01		0.99	
204-5p	15						0.24			0.39		0.08	0.79		0.05		0.85		0.45		0.09		0.12		0.67	
**223-3p**	20						0.37			0.11		**0.46**	**0.04**		0.26		0.28		0.18		0.44		0.28		0.24	

R_s_ Spearman correlation coefficient. WBV, whole brain volume; CGMV, Cortical grey matter volume; WMV, White matter volume T1, T1 lesion volume. Statistically significant associations are highlighted in bold.

## Discussion

Several microRNAs have been associated with MS in many articles. Most of these studies are very heterogeneous and mainly describe differences in MS versus controls, or in different stages (remission versus relapses) or phenotypes of the disease (relapsing versus progressive). There are fewer studies focusing on the association of miRNAs with clinical or radiological variables (8, 11, 17, 33), and only one of them combining EDSS, cognitive functioning and MRI together, but in pediatric population (19). In our work we analyzed the relationship of a preselected microRNAs through topological analysis with both clinical, cognitive and MRI variables in adult MS.

GA is a synthetic polypeptide made of the random combination of 4 amino acids, similar to the myelin basic protein (34). Its mechanism of action is not completely understood, but it is assumed that it binds to the HLA-II complex regulating the immune response at several cellular processes (35). Thus, different patterns of miRNA, as well as changes in miRNA expression related to its immunomodulatory effects, could modify the therapeutic response to this drug. There are several articles describing the miRNA changes associated with other disease modifying treatments such as IFN (36–40), natalizumab (41–44), dimethyl fumarate (45, 46) or fingolimod (47–51), but less information is available regarding GA (52, 53). In one article that analyzed the miRNAs changes in a mouse-EAE model treated with GA, the authors found miR-155.5p, miR-27a.3p, miR-9.5p and miR-350.5p as putative GA-treatment response biomarkers (52), all of which were associated with an altered polarization of T cells toward a Th1 and Th17 phenotypes. In the other study, with MS patients, a change in miR-146a.5p and miR-142.3p after GA treatment was found, both of those miRNAs related to immunotolerance *via* increasing the suppressor function of T regulatory cells (53).

In our article, we notably found a correlation between GA and two of those previous microRNAs. And more interestingly they were associated with more than one clinical or radiological variable. They were miR-146a.5p (correlated with EDSS and SDMT), and mir-9.5p (correlated with EDSS and thalamic volume, and with a trend to association with SDMT).

MicroRNA-155.5p was investigated in our study, but we did not find any correlation with the clinical or radiological variables. The other reported microRNAs were not included in this research as they were not preselected in the 20 best candidates made by the simple topological analysis (Anaxomics^®^).

MicroRNA 9.5p has important functions in the regulation of immune responses. It appears to promote inflammatory responses inducing Th17 cells and microgial activation through different mechanisms (54). In this way, specific blockage of miR-9.5p has been suggested as a potential therapeutic strategy for treating different neuroinflammatory conditions (55). In fact, as previously reported, in an Experimental Allergic Encephalomyelitis (EAE) model of MS, miR-9.5p was increased at the peak of the disease, and its levels were reduced with GA treatment (52). In our study the association of elevated levels of miR-9.5p with higher EDSS and thalamic atrophy reinforce this possible pathogenic effect of miR-9.5p.

MicroRNA 146a.5p has been previously associated with the response to GA (53). In that article miR-326, miR-155, miR-146a.5p and miR-142.3p were aberrantly expressed in peripheral blood mononuclear cells from RRMS patients compared to controls. This pattern did not changed in IFN-b treated patients, but miR-146a.5p and miR-142.3p were significantly reduced after GA treatment. MicroRNA-146a.5p is an important regulator of the immune system and seems to participate in the suppressor function of T regulatory cells (56), down-regulation of Th17 cells (57) and promotion of M2 (immunosuppressive) polarization of macrophages (58). Given these immunotolerance functions, the increase of miR-146a.5p in MS could be related to an indirect mechanism to try to counterbalance the inflammatory state in these patients rather a direct effect of miR-146a.5p in MS pathology. The association of higher levels of this microRNA with worse EDSS and SDMT outcomes would mean a higher MS activity and a worse prognosis rather than a direct pathogenic effect.

MicroRNA-223.3p has been shown to have neuroprotective effects in an animal model of MS. It seems that miR-223.3p could exert its functions by blocking the glutamate receptor signaling. In human studies it has been shown a higher expression of this miRNA in MS versus controls (59), as well as in relapses versus remission and in RRMS versus PPMS (60). For these reasons it has been postulated that miR-223.3p would be upregulated as a compensatory mechanism in response to inflammation, and would exert a direct neuroprotective effect by reducing excitotoxicity. These data are in line with the protective effect found in our study, but they do not allow us to extract any conclusions about it utility as clinical biomarker.

Even though the strongest associations were found with miR-126.3p and miR-200c.3p, less data is available regarding these microRNAs and miR-138.5p. MicroRNA 126.3p has been linked on the one hand with fibrotic responses (61) and on the other hand with clinical response in other autoimmune diseases (62). In MS it has been shown to be upregulated during the remission phase of the disease (60). In this regard it could be postulated as having a protective effect in MS. But this data is in contrast with the supposed pathogenic effect found in our work, and there are not other data to support any implication of mir-126.3p in MS pathology. Finally, miR-200c.3p and miR-138.5p seem to regulate apoptosis and cell proliferation in different forms of cancer, but they have not been previously related to multiple sclerosis either, and, as with mir-126.3p, there are not sufficient data to elaborate any conclusions about the findings of our study. It would be very interesting to further evaluate these microRNAs to confirm these associations.

One limitation of our study was the small number of patients. But the oriented pre-selection of microRNAs made by the topological analysis described in the methods could have overcome this problem. This procedure would allow to minimize the sample to bring out significant clinical relationships, and it would explain the meaningful and high number of statistical associations found in our study.

In conclusion, these data support the hypothesis of miRNA as potential biomarkers in this disease. More studies are needed, with bigger samples, controls and longitudinal designs to validate these results and to better understand the role of miRNAs in the pathogenesis, monitoring and therapeutic response of MS.

## Data Availability Statement

The original contributions presented in the study are included in the article/Supplementary Material. Further inquiries can be directed to the corresponding author.

## Ethics Statement

The studies involving human participants were reviewed and approved by Getafe University Hospital. The patients/participants provided their written informed consent to participate in this study.

## Author Contributions

MD-M: conceptualization (equal), formal analysis (equal) investigation (equal), writing-Original draft preparation (equal); IC: conceptualization (equal), formal analysis (equal), funding Acquisition (equal), Investigation (equal), writing-Original draft preparation (equal); LT: conceptualization (equal), data curation (equal), investigation (equal), resources (equal), writing-review and editing (equal); YA: Data curation (equal), Investigation (equal), Resources (equal), Writing-review and editing (equal); SP-P: Investigation (equal), Resources (equal), Writing – review and editing (equal); AM: Investigation (equal), Resources (equal); PG: Data curation (equal), Resources (equal), SA: Data curation (equal), Resources (equal); EA: Investigation (equal), Resources (equal); CL: Conceptualization (equal), Funding acquisition (equal), Investigation (equal), Writing – Original draft preparation (equal), Supervision (equal); RA-L: Conceptualization (equal), Investigation (equal), Writing – Original draft preparation (equal); Supervision (equal). All authors contributed to the article and approved the submitted version.

## Funding

The authors disclosed receipt of the following financial support for the research, authorship, and/or publication of this article: This work was supported by an Investigator Sponsored Research Grant by TEVA [TV-44400-CNS-60250]; by AELEM (Spanish Association for the fight against Multiple Sclerosis); by the Torrejon University Hospital; and by Fundación LAIR. These supporting sources did not have any involvement or restrictions regarding this publication.

## Conflict of Interest

IC declares: having received payments as speaker, and support for attending meetings from Bayern, Biogen, Merck, Novartis, Roche Sanofi and Teva. YA declares: has received funding for research projects or in the form of conference fees, mentoring, and assistance for conference attendance from: Bayer, Biogen, Roche, Merck, Novartis, Allmirall and Sanofi-Genzime.

The remaining authors declare that the research was conducted in the absence of any commercial or financial relationships that could be construed as a potential conflict of interest.

## Publisher’s Note

All claims expressed in this article are solely those of the authors and do not necessarily represent those of their affiliated organizations, or those of the publisher, the editors and the reviewers. Any product that may be evaluated in this article, or claim that may be made by its manufacturer, is not guaranteed or endorsed by the publisher.
